# An update on the distribution of *Glossina* (tsetse flies) at the wildlife-human-livestock interface of Akagera National Park, Rwanda

**DOI:** 10.1186/s13071-021-04786-3

**Published:** 2021-06-02

**Authors:** Richard S. Gashururu, Samuel M. Githigia, Methode N. Gasana, Richard Habimana, Ndichu Maingi, Giuliano Cecchi, Massimo Paone, Weining Zhao, Daniel K. Masiga, James Gashumba

**Affiliations:** 1 School of Veterinary Medicine, University of Rwanda, P.O. Box 57, Nyagatare, Rwanda; 2 Faculty of Veterinary Medicine, University of Nairobi, P.O. Box 29053, Nairobi, Kenya; 3 Rwanda Agriculture and Animal Resources Board, PO. Box 5016, Kigali, Rwanda; 4 Food and Agriculture Organization of the United Nations (FAO), Animal Production and Health Division, Rome, Italy; 5 International Centre of Insect Physiology and Ecology (Icipe), P.O. Box 30772-00100, Nairobi, Kenya; 6 Rwanda Polytechnic, P.O. Box 164, Kigali, Rwanda

**Keywords:** *Glossina*, Distribution, Trypanosomosis, Wildlife-human-livestock interface, Akagera NP, Rwanda

## Abstract

**Background:**

*Glossina* (tsetse flies) biologically transmit trypanosomes that infect both humans and animals. Knowledge of their distribution patterns is a key element to better understand the transmission dynamics of trypanosomosis. Tsetse distribution in Rwanda has not been well enough documented, and little is known on their current distribution. This study determined the current spatial distribution, abundance, diversity, and seasonal variations of tsetse flies in and around the Akagera National Park.

**Methods:**

A longitudinal stratified sampling following the seasons was used. Biconical traps were deployed in 55 sites for 6 consecutive days of each study month from May 2018 to June 2019 and emptied every 48 h. Flies were identified using FAO keys, and the number of flies per trap day (FTD) was used to determine the apparent density. Pearson chi-square (*χ*2) and parametrical tests (t-test and ANOVA) were used to determine the variations between the variables. The significance (*p* < 0.05) at 95% confidence interval was considered. Logistic regression was used to determine the association between tsetse occurrence and the associated predictors.

**Results:**

A total of 39,516 tsetse flies were collected, of which 73.4 and 26.6% were from inside Akagera NP and the interface area, respectively. Female flies accounted for 61.3 while 38.7% were males. Two species were identified, i.e. *G. pallidipes* [*n* = 29,121, 7.4 flies/trap/day (FTD)] and *G. morsitans centralis* (*n* = 10,395; 2.6 FTD). The statistical difference in numbers was significant between the two species (*p* = 0.000). The flies were more abundant during the wet season (15.8 FTD) than the dry season (4.2 FTD). Large numbers of flies were trapped around the swamp areas (69.1 FTD) inside the park and in Nyagatare District (11.2 FTD) at the interface. *Glossina morsitans* was 0.218 times less likely to occur outside the park. The chance of co-existing between the two species reduced outside the protected area (0.021 times).

**Conclusions:**

The occurrence of *Glossina* seems to be limited to the protected Akagera NP and a narrow band of its surrounding areas. This finding will be crucial to design appropriate control strategies. *Glossina pallidipes* was found in higher numbers and therefore is conceivably the most important vector of trypanosomosis. Regional coordinated control and regular monitoring of *Glossina* distribution are recommended.

**Graphic Abstract:**

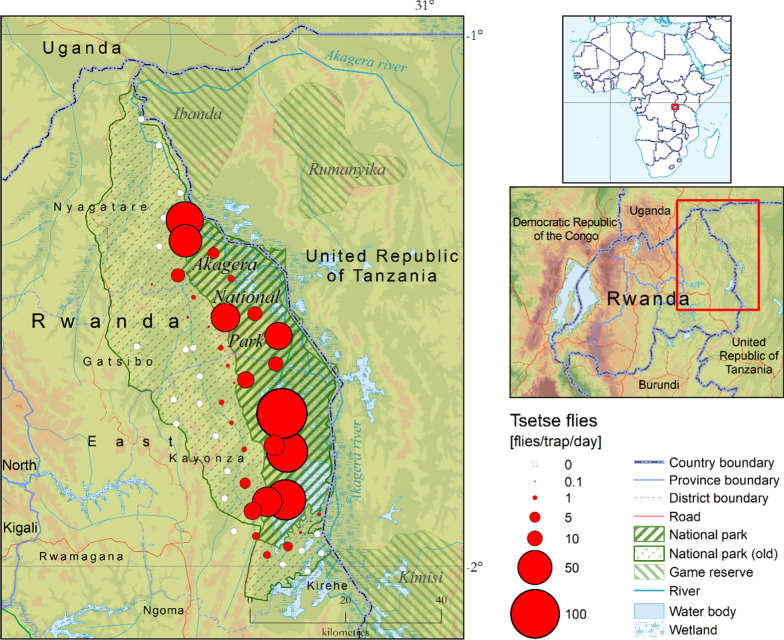

**Supplementary Information:**

The online version contains supplementary material available at 10.1186/s13071-021-04786-3.

## Background

*Glossina* (tsetse flies) are biological vectors of trypanosomes, which cause trypanosomiasis in both humans and animals through blood-feeding [1–4]. The distribution patterns of tsetse species in an area is a key element to better understand the transmission dynamics of trypanosomosis [5, 6]. This information is a requirement for strategic, risk-based control of the vectors and the disease [7, 8].

The presence and abundance of *Glossina* are associated with the environment [9]. The habitat, land use, and ecological settings are determinants of tsetse fly distribution and therefore the disease transmission [10, 11]. In addition, the availability of hosts and their distribution in an area determine the dispersal of tsetse flies, especially the savannah group [9].

Environmental changes play a role in changing disease transmission [12]. Habitat fragmentation and human activities reduce the distribution and abundance of savannah species considerably, leading them to be confined to protected areas where they find a conducive environment and hosts to feed on [13–15]. However, this situation results in an increased challenge of tsetse bites on livestock and humans around the protected infested area [12].

Information on *Glossina* distribution and trypanosomes in Rwanda was much documented during the colonial period [16–20]. However, little has been done to update this information since independence [21].

Three species of tsetse flies, i.e. *Glossina pallidipes* (subgenus *Glossina* (*morsitans*) group—Austen 1903), *G. morsitans centralis* (subgenus *Glossina* (*morsitans*) group—Machado 1970), and *G. brevipalpis* (subgenus Austenina (*Fusca*) group—Newstead 1910) were reported in the lowland eastern savannah region of Rwanda [20, 22, 23]. The fourth species, *G. fuscipes martinii* (subgenus Nemorhina *(palpalis)* group—Zumpt, 1935), was once reported in the south-west region of Rwanda bordering Burundi [24]. However, the species has not been reported again since then [25]. The Kagera area is comprised of the former Mutara region and the affiliated protected areas including Akagera NP. Population growth has over time reduced the suitable habitats for *Glossina* because of land acquisition [26]. The de-gazettement of roughly two-thirds of the protected land comprising the entire Mutara hunting area (300 km^2^) and part of Akagera National Park for resettlements and farming activities after the year 1997 greatly reduced the protected areas [27, 28]. This reduced the Akagera NP to about 1120 km^2^ out of the 2500 km^2^ initially gazetted [28, 29].

**Fig. 1:**
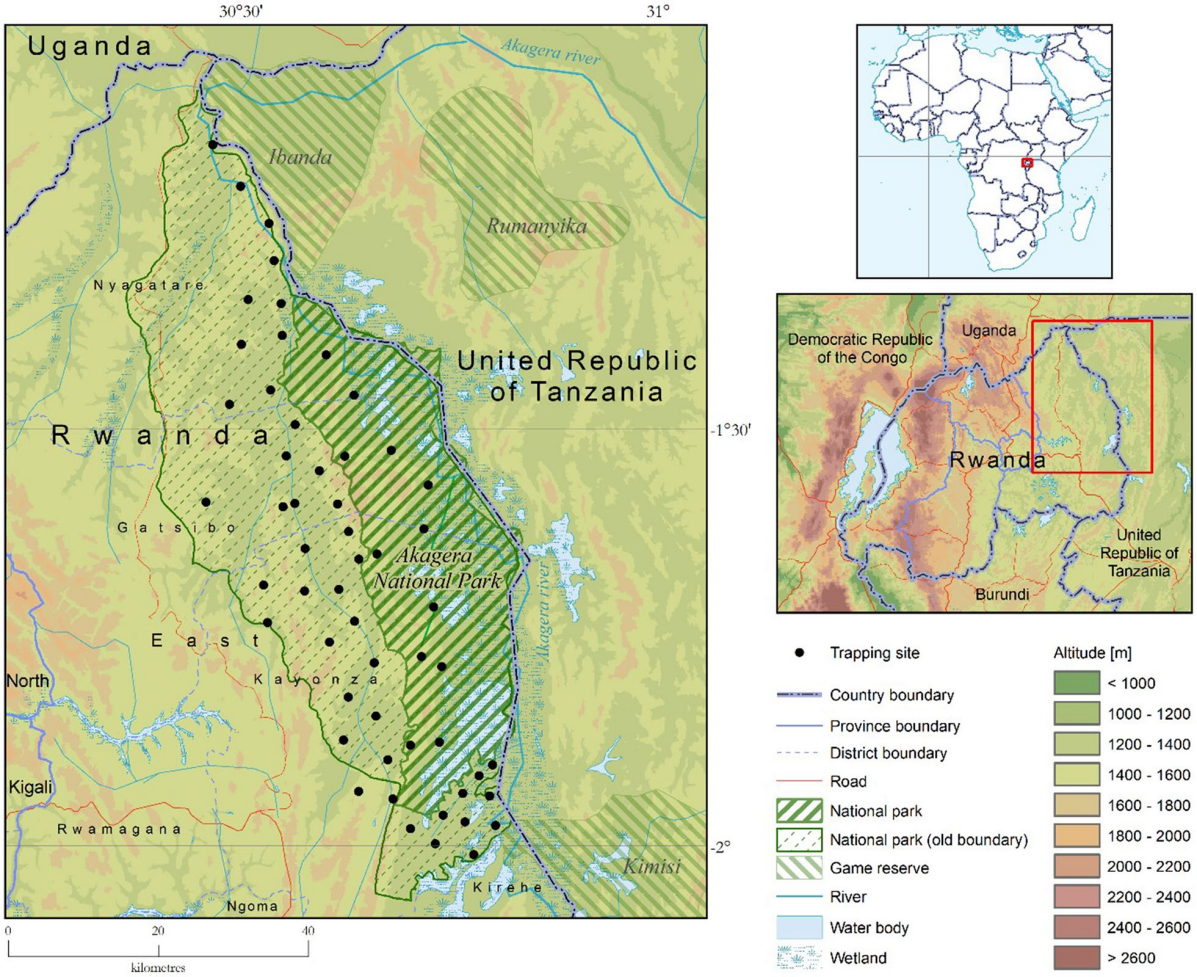
Study area with the old park boundaries

Akagera NP is adjacent to other game reserves in Tanzania (Ibanda Game Reserve in the north and Kimisi Game Reserve in the south) (Fig. 1). Akagera NP is a known home to tsetse flies, and its surroundings are not spared by tsetse challenge and the transmission of trypanosomosis. The park has suitable habitats for tsetse survival and hosts a high concentration of preferred wild animals such as warthogs and buffaloes on which the flies constantly feed [30]. Along the park boundary, many cattle farms surround its entire length together with the settled farmers homesteads [31]. This situation makes the area a suitable habitat and therefore increases the tsetse challenge at the interface. The strategic control of diseases that may originate from wildlife or which are shared among livestock, wildlife and humans at the interface is crucial in the area [32].

Tsetse-transmitted trypanosomiasis is endemic in the same region. The savannah tsetse species believed to be efficient vectors of human and animal infective trypanosomes are prevalent in Akagera ecosystem [21]. AAT is reported by farmers in the regions surrounding the park [33, 34], and the trypanosome infections have been detected in tsetse flies and cattle [21]. Few sporadic cases of *Trypanosoma brucei* *rhodesiense* sleeping sickness were reported in Rwanda around 1990 [35]. However, no recent positives cases have been reported [36–38]. This study determined the current spatial distribution, abundance, diversity, and seasonal variations of *Glossina* species in the region. This information is crucial in order to design evidence-based strategies for the control of the vector and tsetse-transmitted trypanosomosis [7, 39], and it particularly contributes valuable entomological data to validate the elimination of Human African Trypanosomosis (HAT) [37].

## Methods

### Study area

The study was carried out in the eastern province of Rwanda, where *Glossina* are still reported in lowlands characterized by a depressed relief. The province shares borders with Tanzania in the east and Uganda in the north (Fig. 1).

The study focused on Akagera NP and its surroundings, and it extended over three neighboring districts (i.e. Kayonza, Gatsibo, and Nyagatare), with altitudes ranging between 1200 and 1700 m. The interface area outside the Akagera NP is dominated almost entirely by cattle farms. The rains follow a bimodal pattern with an average rainfall of about 1000 mm per annum, even though it is less regular compared to other regions of the country, leading to frequent dry spells. At the interface, the vegetation cover consists of grassland, woodland, and often bushland closer to the park especially for localities in which the study was conducted. The temperature varies between 19 and 29 °C. The long dry season spans the months of June and July up to August while the long wet season comprises the months of March, April, and May [40].

Established as a national park in 1934, the park draws its name from the Akagera River that runs along the international border with the United Republic of Tanzania [30]. It is the only protected savannah region in Rwanda and therefore the only shelter for savannah-adapted species of tsetse. Akagera NP has a sub-arid savannah habitat, subdivided into three ecosystems: swamp (wetland and wetland fringes), mountain, and savannah. There are different vegetation types within the above ecosystems, mainly grasslands, bushed grasslands, wooded grasslands, woodland (mainly Acacia), and dry and humid forests [30, 41]. The wetlands are permanently flooded areas with marshlands and a complex system of lakes fed by the Akagera River. The wetlands cover 30% of the park surface, and the terrestrial part makes up the remaining 70%. The altitude is characteristic of the eastern region; however, it has a mountainous north to south-central ridge along the western boundary that can reach nearly 1750 m [42].

### Entomological survey

A longitudinal stratified sampling was carried out from May 2018 to June 2019 to determine seasonal variations in tsetse fly populations as described by Leak and Vreysen [43]. Inside the park, ecosystems were used for the stratification of sampling, whereas the districts were used at the interface area outside the park. In each of three park ecosystems (i.e. swamps, savannah, and mountains), locations were randomly selected; nonetheless, the most suitable sites for tsetse were purposefully chosen. At the interface, in addition to stratification using the districts, the same approach was used to select the sites. The information on the potential *Glossina* habitat suitability such as near human residences, the density and distribution of the cattle population, communal watering points, grazing areas, overnight cattle collection, and the farmers’ knowledge on the existence of flies was taken into account. Fifty-five sites (12 inside the park and 43 along the interface) were therefore selected for the study. To determine the effect of the distance to the park at the interface, sites located < 3 km from the park border were considered as close, while those located > 3 km were considered as distant.

Biconical traps [44] supplied by Vestergaard Frandsen (Lausanne, Switzerland) were used in the study. These traps are widely used and are efficient in sampling and population monitoring [45]. Traps were deployed for 6 consecutive days of each month (3 months in the rainy season and 3 months in the dry season) and emptied every 48 h [46]. Two traps were deployed in the same site at a distance of 200 m [6], and the fly catches of the two traps were later combined to represent a site. The number of traps deployed in area was determined by its size [47]. The trap effectiveness was improved by using a 3-week-old cow urine and acetone as bait, kept in plastic bottles with an opening dispensing the odor. Grease was applied at the bottom of the trap support to prevent ants from climbing into the trap. Each trapping site was georeferenced by a global positioning system device (Garmin Ltd, Olathe, KS, USA) to generate a map later.

Flies were morphologically identified using a stereomicroscope (Opta Tech SK392, Poland) as described in the FAO training manuals [43, 48]. For each site, records of the number, species, sex, and other biting flies were taken. Flies with damaged or lost body parts were excluded from the identification and are not part of the results.

### Analysis

The analysis was performed by SPSS software (SPSS Inc., IL, USA). The average number of flies caught per trap per day (FTD), referred to as the apparent density (AD), was obtained after dividing the total number of flies caught in a trap by the number of days the trap has been in place. The term ‘abundance’ refers to the apparent density to express the average number of flies available in a specific site [6]. Pearson chi-square (*χ*2) was used to determine the variations between the variables such as the area, season, district, species, sex, and localities. A *P*-value < 0.05, significant at the 95% confidence interval, was taken into account. Parametrical tests (*t*-test and ANOVA) were used to compare whether the average number of flies caught per trap per day differed between factors/predictors such as area, season, ecosystem, month, district, and locality. Logistic regression was used to determine the association between the occurrence of tsetse flies and the associated predictors (cited above and explained in the design), followed by the determination of odds ratios (OR) for each predictor.

## Results

**Fig. 2:**
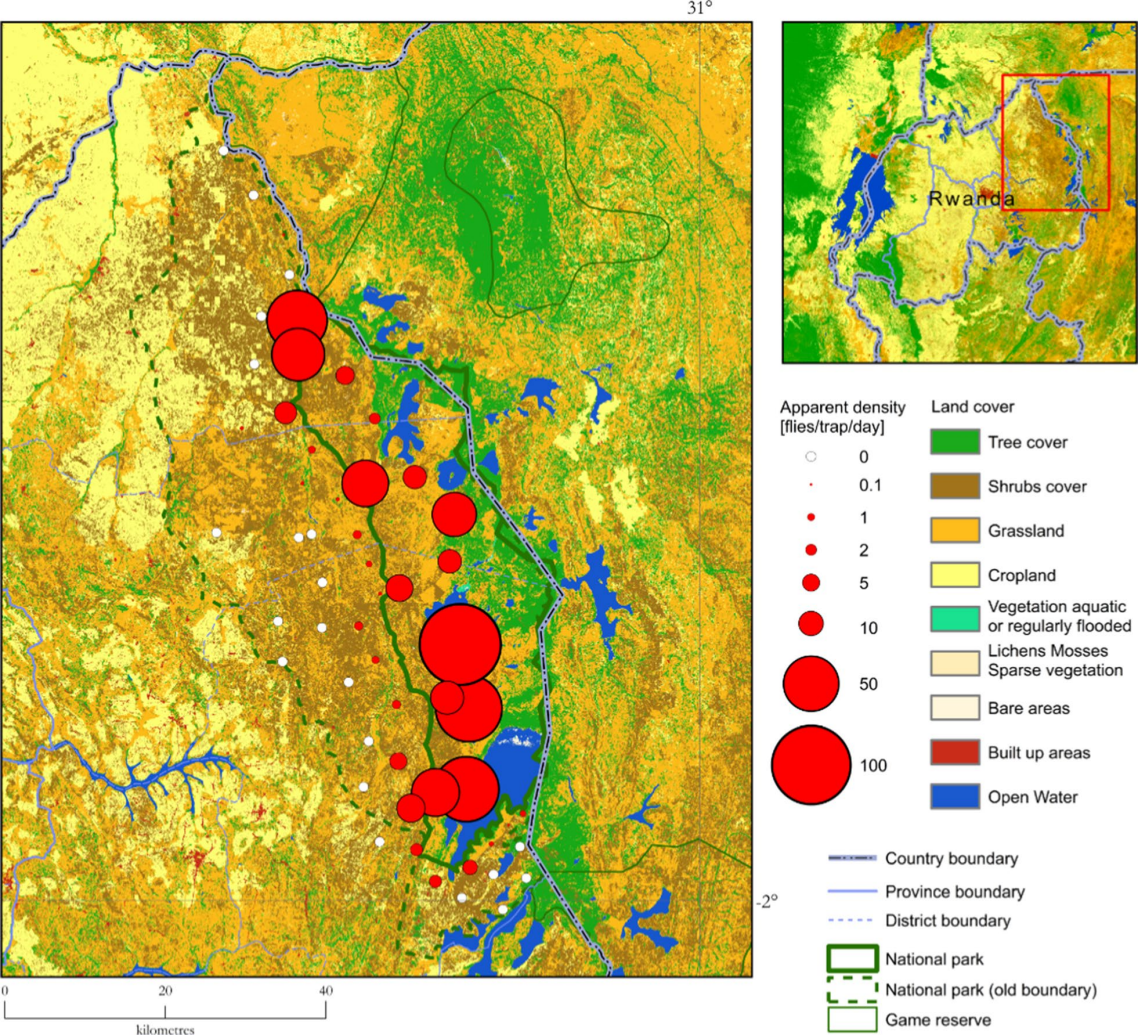
Apparent density with land cover (seasons and species combined)

**Fig. 3:**
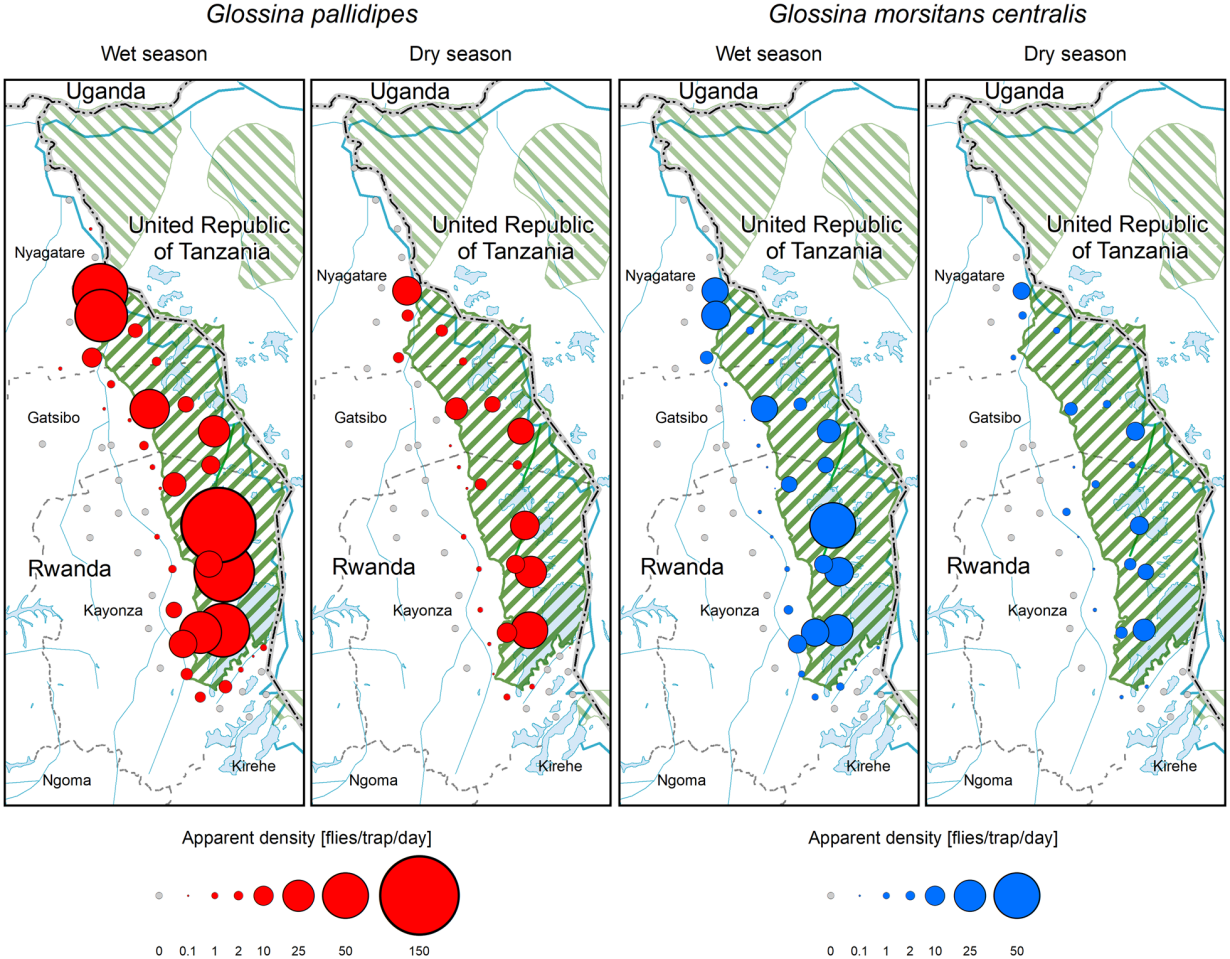
Seasonal occurrence of *G. pallidipes* and *G. morsitans centralis* in and around Akagera National Park. The area of the circles is directly proportional to the apparent density

**Fig. 4:**
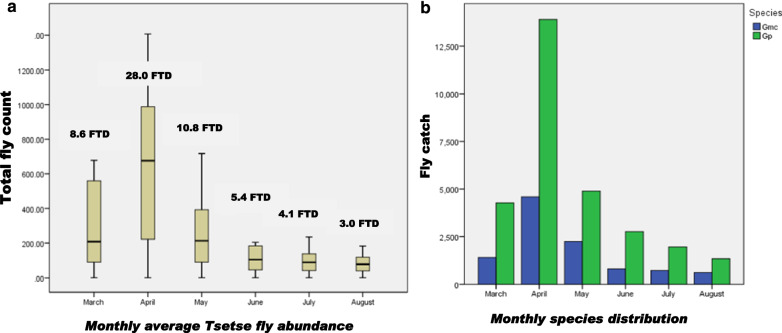
Monthly fly abundance (**A**) and species composition (**B**). The difference is highly significant in April (*P* = 0.00, 95% CI) compared to other months. *Gmc*  *Glossina morsitans centralis*, *Gp* = *Glossina pallidipes*, *FTD* fly per trap per day

A total of 39,516 tsetse flies was collected, of which 29,019 (73.4%) and 10,497 (26.6%) were from Akagera NP and the interface area, respectively (Fig. 2). The difference in the occurrence of tsetse flies between the two areas was statistically significant (*p* = 0.000). Two species of *Glossina*, *G. pallidipes* (*n* = 29,121; 7.4 overall FTD) and *G. morsitans centralis* (*n* = 10.395; 2.6 overall FTD) were identified, of which the females accounted for 61.3% (*n* = 24,225), while 38.7% (*n* = 15,291) were males (Table 1). The difference in occurrence of the two species was statistically significant (*P* = 0.000). The flies were more abundant during the wet season (*n* = 31,295; 15.8 FTD) compared to the dry season (*n* = 8221; 4.2 FTD). Figures 3 and 4 show the seasonal distribution in densities and species with details of the respective months.

**Table 1:** Distribution of *Glossina* by species and sex across study areas and seasons

Variable	*Glossina morsitans centralis*			*Glossina pallidipes*			Grand total	Overall FTD	*p* value
	M	F	Σ	M	F	Σ			
Dry season	786	1380	2166	2288	3767	6055	8221	4.2	
Wet season	3250	4979	8229	8967	14,099	23,066	31,295	15.8	< 0.000
Interface	894	1485	2379	3114	5004	8118	10,497	3.4	
Akagera NP	3142	4874	8016	8141	12,862	21,003	29,019	33.6	< 0.000

*M* male, *F* female, *Σ* total, *FTD* flies/trap/day

**Fig. 5:**
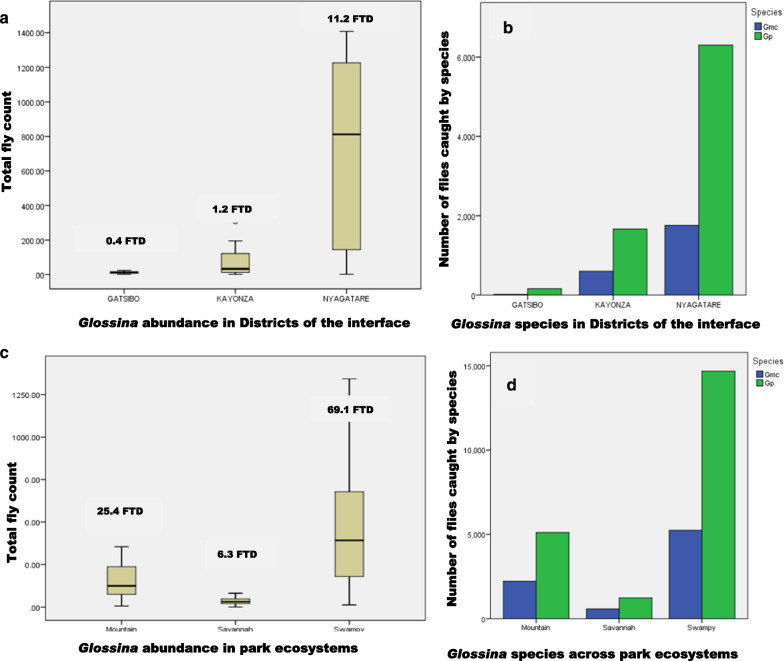
The abundance and distribution of *Glossina* within the study strata. Districts are strata at the interface (**A**, **B**) and ecosystems of Akagera NP (**C**, **D**): *Gmc* *Glossina morsitans centralis*, *Gp* *Glossina pallidipes*, *FTD* fly per trap per day

**Fig. 6:**
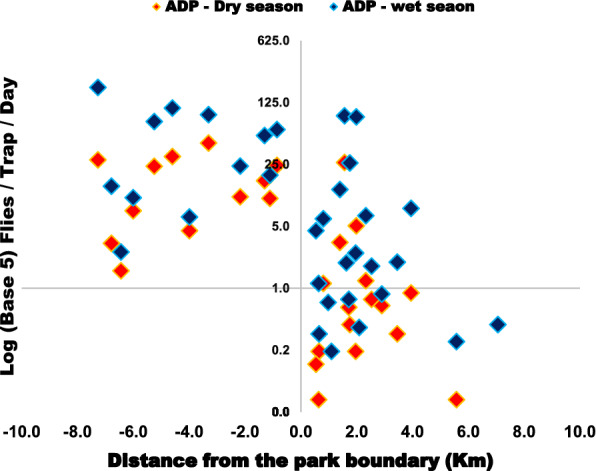
Tsetse density with reference to the distance from the park boundary

Inside the park, the fly distribution differed between ecosystems, where a higher fly catch was recorded from the swamps area (*n* = 19,904; 69.1 FTD) compared to the mountains (*n* = 7304; 25.4 FTD) and the savannah (*n* = 1,811; 6.3 FTD). The difference in fly catch between ecosystems was statistically significant (*P* = 0.000). Variations in fly catch were observed outside the park, within the three districts surrounding the park. Tsetse catch was *n* = 8053 (11.2 FTD), *n* = 2266 (1.2 FTD), and *n* = 178 (0.4 FTD), respectively, for Nyagatare, Kayonza, and Gatsibo (Fig. 5). The difference in catch was statistically significant (*P* = 0.000) when Kayonza and Gatsibo were compared to Nyagatare, but not significant between Gatsibo and Kayonza (*P* = 0.107). The occurrence of *Glossina* at the interface area drops rapidly in a distance of a few kilometers from the park boundary (Fig. 6). The probability of co-existence between *G. pallidipes* and *G. morsitans centralis* reduced outside Akagera NP (Table 2).

**Table 2:** Multivariate logistic regression analysis for occurrence and co-occurrence of tsetse species

Predictor	*G. pallidipes*			*G. morsitans*			Coexistence		
	Fly catch	OR (95% CI)	*p*	Fly catch	OR (95% CI)	*p*	Fly catch	OR (95% CI)	*p*
Area									
Akagera N.P	21,003	1.00		8016	1.00		29,019	1.00	
Interface	8118	1.275 (1.216–1.336)	0.000	2379	0.218 (0.113–0.423)	0.000	10,497	0.021 (0.007–0.065)	0.000
Season									
Dry	6055	0.899 (0.858–0.942)	0.000	2166	1.412 (1.263–1.578)	0.000	8221	2.860 (1.781–4.593)	0.000
Wet	23,066	1.00		8229	1.00		31,295	1.00	
Month									
March	4275	1.416 (1.264–1.586)	0.000	1399	0.648 (0.469–0.897)	0.009	5674	0.312 (0.163–0.596)	0.000
April	13,903	1.512 (1.365–1.675)	0.000	4589	0.547 (0.432–0.691)	0.000	18,492	0.002 (0.001–0.004)	0.000
May	4888	1.093 (0.980–1.219)	0.111	2241	0.670 (0.515–0.872)	0.003	7129	0.023 (0.011–0.48)	0.000
June	2763	1.628 (1.437–1.843)	0.000	812	0.507 (0.375–0.685)	0.000	3575	0.073 (0.034–0.153)	0.000
July	1955	1.283 (1.128–1.459)	0.000	733	0.664 (0.486–0.907)	0.010	2688	0.070 (0.27–0.180)	0.000
August	1337	1.00		621	1.00		1958	1.00	
Ecosystem									
Mountain	5097	0.823 (0.775–0.873)	0.000	2207	1.215 (1.145–1.289)	0.000	7304	1.000 (0.00 -.^b^)	1.000
Savannah	1231	0.756 (0.582–0.839)	0.000	580	1.322 (1.192–1.467)	0.000	1811	ND	ND
Swampy	14,675	1.00		5229	1.00		19,904	1.00	
District									
Kayonza	1663	2.335 (1.447–3.769)	0.001	603	0.428 (0.265–0.691)	0.001	2266	99.360 (63.481–155.517)	0.000
Gatsibo	159	0.770 (0.691–0.857)	0.000	19	1.299 (1.167–1.446)	0.000	178	13.090 (9.096–18.836)	0.000
Nyagatare	6296	1.00		1757	1.00		8053	1.00	

Reference categories: Akagera NP for area, Wet for seasons, August for months, Swampy for ecosystems, Nyagatare for districts, and Yes for co-existence

*OR* odds ratio, *CI* confidence interval, *ND* not determined

High catches were mainly found inside the park and a narrow band of Nyagatare District. High tsetse densities are associated with swampy vegetation.

*Glossina* were more abundant in Akagera NP than at the interface, and the wet season was associated with high fly catches compared to the dry season. Note the variation in sex composition across seasons and study areas. The female flies were more abundant than the males.

Negative distance values signpost the sites inside Akagera park, whereas positive values show the sites at the interface area. The further tsetse fly was captured 7 km from the park boundary during the wet season.

### Maximum likelihood analysis for the existence and co-existence of *G. pallidipes* and *G. morsitans centralis*

The chance of co-existing between the two species reduced outside the protected area (0.021 times). The probability to get *G. morsitans centralis* was 1.412 times more in the dry season than *G. pallidipes* (0.899 times). The coexistence of both species reduced 2.860 times more in the dry season than during the wet season. With reference to August, more *G. pallidipes* were collected in June (1.628 times), April (1.512 times), and March (1.416 times).

Inside the park, *G. pallidipes* occurred less in the mountain (0.823 times less) and savannah ecosystems (0.756 times less) than in swampy areas. However, *G. morsitans centralis* increased 1.215 times more in the mountains and 1.322 times more in the savannah. The co-existence of both species was the same in the mountains and swamps. In Nyagatare District, *G. pallidipes* occurred 2.335 times more in Kayonza and 0.770 times less in Gatsibo. The occurrence of *G. morsitans centralis* was 0.428 times less in Kayonza and 1.299 times more in Gatsibo. The co-existence of both species reduced 13.090 times in Gatsibo and enormously in Kayonza (99.360 times) compared to the situation in Nyagatare.

## Discussion

This study shows an important shrinkage of tsetse distribution outside the current park as opposed to the old distribution maps [20, 21, 24]. One of the major reasons for this is that most of the previously reported infested area and half of Akagera Park were rezoned for human settlement and farming activities [27, 28]. This has led to changes in land use, habitat break-up, and increased human activities, which in turn reduce tsetse distribution in an area, especially for species of the savannah group [10, 14].

This situation has been the norm for many areas in sub-Saharan Africa, where the edges of protected areas are experiencing demographic pressure [49]. A similar reduction in *G. morsitans* populations was reported in eastern and southern Africa, including Zambia [13, 50] and Malawi [51]. The lack of suitable habitats across the interface could explain this decline around Akagera NP [14]. This pattern is also apparent in western Africa, where tsetse flies of the morsitans group are increasingly found only in remnant populations, mostly associated with protected areas [15, 52].

In a survey conducted in 2012 in Tanzania, the game reserves of Ibanda and Kimisi were found to be infested with *Glossina morsitans* and characterized as high-risk tsetse-infested areas. The same survey characterized an area neighboring Kimisi, a game-controlled area, as a non-tsetse infested area, possibly because of a lower concentration of wild animals, the source of blood meals for tsetse flies [53]. All three areas are at the border with Rwanda and adjacent to Akagera NP. The linkage between the protected areas in Tanzania and Akagera NP in terms of tsetse infestation is a reminder of the transboundary nature of the trypanosomosis problem.

This study clearly shows that the Akagera NP remains a favorable refuge for tsetse populations in the area, therefore contributing to the constant risk of trypanosomosis transmission in the neighborhood. Tsetse habitat in the eastern region of Rwanda has reduced greatly because of demographic pressure. The remaining savannah habitat seems to favor *G. pallidipes*, which tends to dominate other savannah species in the region [54–56]. The high tsetse abundance in Akagera NP was found all year round with seasonal variations from the wet to dry season. The abundance is highly associated with the Acacia swampy ecosystem as demonstrated in Tanzania by Ngonyoka [6].

The tsetse challenge to livestock and humans remains at the interface with the park where the community of farmers is settled and many cattle are reared. Van den Bossche [12] described a similar scenario, and the same was found around Serengeti National Park in Tanzania [14]. The distance to the Akagera NP boundary, type of vegetation, and land use seemed to be the factors determining the abundance of tsetse flies at the interface area as it was confirmed by Salekwa [57]. Among the three strata used at the interface, many *Glossina* were observed in Nyagatare District. The latter district is located in the north of the park where there is a high concentration of wild animals [30] and high densities of livestock, which contribute to making the area a more suitable tsetse habitat.

The higher abundance of tsetse flies during the rainy season is explained by their behavioral activities. Tsetse flies customarily aggregate in dense vegetation in the dry season and disperse remarkably into areas that are more open during the rainy season [43, 58]. This behavior affects the fly catches [46], one of the reasons why traps deployed during the sunny period (June, July, and August) caught fewer flies than those deployed in the wet season (March, April, and May) according to the local climatic conditions. Nevertheless, the combined effects of hosts, vegetation, climate, and human settlements affect the abundance and distribution of flies.

This study only found two species, G. *pallidipes* and *G. morsitans centralis*, among the three previously reported species. Their distribution is fairly homogeneous for both species across all the vegetation types. *G. pallidipes* was numerically dominant in all surveyed localities. It had densities three times higher than *G. morsitans centralis*, but this was not reflected by a broader distribution. Most traps that captured zero *G. morsitans centralis* also did not catch any *G. pallidipes*. There was a relatively higher population of *G. morsitans centralis* in this study compared to earlier findings of Mihok [21]. *G. pallidipes* were *82.6*% whereas *G. morsitans centralis* were 15.2%. However, the later work was area-limited and did not include Akagera NP, comparisons may, therefore, be misleading. In particular, the difference could be linked to the adjacent Tanzanian side, which was reported to be infested with *G. morsitans* [53], though the traps in these areas did not catch any of these during this study.

The abundance of *G. pallidipes* is in line with the situation in several other countries of the eastern and southern Africa regions [53, 56, 59]. This species is the most widely distributed tsetse species in those regions and hence is the main vector of AAT and potentially the vector of rhodesiense sleeping sickness [60]. However, recent studies highlight the genetic modifications amid *Glossina* populations [61] due to environmental changes [62–64], which could lead to the dominance and adaptations of certain species in a region. This needs to be assessed in the Akagera region as well to locate isolated tsetse populations. This knowledge could help to assess the risk of reinvasion and inform about the feasibility and desirability of eliminating tsetse-transmitted trypanosomes [65, 66].

This study did not find any *G. brevipalpis* though it was previously reported. A study by Mihok [21] found 2.2% in a total fly catch of 312,801 tsetse flies. As stated above, this work was undertaken a few kilometers away and did not include Akagera NP. Another monitoring work done in 2013 by Oloo (unpublished report) in Akagera NP found only one female *G. brevipalpis* in one site of the swamp ecosystem where traps were deployed in this study. It is a forest type species, frequently associated with waterside evergreen thickets and forest islands in savannah habitats. The host preferences are mostly the hippopotamus and the bush pig [67]. This indicates that its conducive habitat in the Akagera region could be the lakeshore swampy forests of the park where it was not found during this survey. The species has both poor response to traps and odor bait attractants. However, *G. brevipalpis* is in most cases found in low numbers when co-existing with the *Morsitans* (savannah) group [68]. There is a need to investigate whether the *morsitans* species hampers the survival and the distribution of this species, especially when it has a conducive environment, or it has simply disappeared from the area.

The absence of tsetse flies in a trap catch does not necessarily mean their absence in the area [9, 69]. Many factors are involved, and the behavior of some species differs in response to the trapping strategy. There is a need for regular monitoring by using different methodologies to better understand the behavior of tsetse populations of the area. In particular, *G. morsitans* responds well to visual moving objects compared to *G. pallidipes*, which is attracted to stationary baits [59, 70, 71].

## Conclusion and potential implications

Both *G. pallidipes* and *G. morsitans* are vectors of trypanosome infections to livestock and humans. From this study, *G. pallidipes* could be the more important vector because of its higher density. Tsetse occurrence seems to be limited to the protected Akagera NP and a narrow band around it. The infestation was significantly lower in the buffer area and dropped quickly to zero at a few kilometer distance away from the park border in both seasons. The decline in tsetse distribution indicates the possibility of controlling the *Glossina* vectors, and eventually African trypanosomosis in the area.

Some control strategies are already being implemented in the area by the national veterinary services such as the distribution of subsidized NGU traps to farmers and sensitization on good farm management. Farmers constantly use trypanocides to fight AAT, but rarely in a systematic way. On the park side, tourists are warned of tsetse flies, and target screens are installed along the roads inside the park. Despite these efforts, a more coordinated control mechanism using a multidisciplinary approach involving all the concerned parties is needed. We suggest a combination of vector control, community engagement, and improved farming practices, as tsetse flies are limited to a known area. The vector control should focus on the spatial targeting of interventions in some areas of the park and the buffer zone around it. The study recommends the regular surveillance of both animal and human trypanosomiasis at the buffer zone even though the tsetse distribution rapidly decreases outside the park. This paper is part of a broader study; data on trypanosomes have been collected and will be presented in a separate paper.

There is a lack of data on tsetse presence in any other area of the country, and there are no records in the veterinary services so far. This leads to hypothesizing that the Akagera region could be the main and possibly the only area with a tsetse challenge in Rwanda. However, there is a need to have a national level picture of the tsetse and trypanosomosis challenge and confirm the absence of the vector from other areas that were historically or are still potentially at risk. Given that the two adjacent game reserves in Tanzania are infested and tsetse flies are dynamic and conducive environment-dependent, we recommend regionally coordinated control efforts and regular monitoring in the predictive areas to keep the distribution updated.

Tsetse data collection was solely based on stationary traps. No fly rounds were carried out, and certain savannah species (i.e. *G. morsitans*) are known to be more attracted by moving targets. We used baited traps only, which may have affected the abundance of some species such as the forest type associated with waterside evergreen thickets and the swamp habitat (Additional files 1 and 2). These species (i.e. *G. brevipalpis*) have a poor response to both traps and baits.

## Supplementary Information

**Additional file 1**: Detailed data set for entomological survey.

**Additional file 2**: Summarized data set for seasonal variation maps.

## Data Availability

The data sets supporting the conclusions of this article are included within the article and its additional files.

## Abbreviations

AAT: Animal African trypanosomiasis
AD: Apparent density
ANOVA: Analysis of variance
CGIAR: Consultative Group on International Agricultural Research
FTD: Fly per trap per day
G.: Glossina
GIS: Geographic Information System
HAT: Human African trypanosomosis
IFAD: International Fund for Agricultural Development
NISR: National Institute of Statistics of Rwanda
NP: National park
OR: Odds ratio
PAAT: Programme Against African Trypanosomosis
PCP: Progressive control pathway
PATTEC: Pan African Tsetse and Trypanosomosis Eradication Campaign
RAB: Rwanda Agriculture and Animal Resources development Board
REMA: Rwanda Environment Management Authority
SPSS: Statistical Product and Service Solutions
UNFAO: Food and Agriculture Organization of the United Nations
WHO: World Health Organisation
